# Disease burden and trends in gout for adolescents from 1990 to 2021, with projections to 2050 globally, in East Asia and China: results from the Global Burden of Disease study 2021

**DOI:** 10.3389/fpubh.2025.1629891

**Published:** 2025-08-13

**Authors:** Lu Yang, Zhenyu Wang, Ligang Cui

**Affiliations:** Peking University Third Hospital, Haidian, China

**Keywords:** gout, adolescent, East Asia, GBD (2021) database, prevalence, disability-adjusted life years (DALYs), age-standardized rates

## Abstract

**Background:**

Gout, a metabolic disorder driven by urate crystal deposition, has been understudied in adolescents, particularly in East Asia and China, where rising incidence aligns with rapid dietary and economic shifts. This study assessed the global, East Asian, and Chinese burden of adolescent gout from 1990 to 2021 and projected trends to 2050.

**Methods:**

Using Global Burden of Disease (GBD) 2021 data, we analyzed incidence, prevalence, and disability-adjusted life years (DALYs) among adolescents aged 10–19 years. Age-standardized rates (ASRs) and estimated annual percentage changes (EAPC) were calculated. Autoregressive Integrated Moving Average (ARIMA) models projected future trends, while smoothing splines explored associations between sociodemographic index (SDI) and DALYs. Risk factor contributions were quantified by gender and region.

**Results:**

From 1990 to 2021, global ASRs of incidence, prevalence, and DALYs rose annually by 0.26%, reaching 109.1, 653.8, and 20.2 per 100,000 in 2021, respectively. East Asia exhibited steeper increases (0.36–0.37% annually), with Taiwan (Province of China) reporting the highest ASRs (prevalence: 1,054.1; DALYs: 33.2 per 100,000). China saw 17.1–25.1% ASR increases, driven by metabolic risks (36.8%), obesity (31.4%), and kidney dysfunction (7.6%). Females consistently bore higher burdens than males across regions. An M-shaped SDI-DALYs relationship peaked at mid-high development levels (SDI ≈ 0.75). Projections indicated stable global trends but rising incidence and prevalence in East Asia and China by 2050.

**Discussion:**

The escalating adolescent gout burden in East Asia and China reflects synergistic effects of obesity, metabolic syndrome, and lifestyle changes. Gender disparities highlight underrecognized risks in females. Sociodemographic transitions initially exacerbate then mitigate gout burden, emphasizing the need for early interventions targeting modifiable risks. Strengthening healthcare infrastructure and gender-specific prevention strategies are critical to curb projected increases in high-risk regions.

## Introduction

Gout is a common and treatable disease caused by the deposition of monosodium urate crystals in articular and non-articular structures (1). The typical first presentation of gout is an intense acute inflammatory arthritis affecting a lower limb joint, accompanied with erythema, limited mobility and fear of physical contact. Delayed diagnosis and Lack of treatments may cause gout to develop into tophi, chronic arthritis and even structural joint damage (2, 3). Gout has a higher prevalence in Australasia and North America, and is more commonly diagnosed in men, with a male-to-female ratio ranging from 2.5 to 11 across different age groups (4). Similarly, in East Asia, the incidence of gout has been increasing in recent years, with a similar gender distribution. In the past two decades, rapid economic development and changes in dietary habits have led to a marked increase in the incidence of gout in China, making it a current disease burden of the Chinese population (5, 6).

Gout in adolescents has long been overlooked, primarily due to a dearth of age-specific data, particularly in developing countries such as China (7, 8). Younger patients tend to have lower treatment adherence, significantly exacerbating the disease burden. As a developing nation with a vast adolescent population and a rising gout burden, China epitomizes the need for comprehensive research in this area. In the context of China and East Asia, assessing the incidence, prevalence, and disability-adjusted life years (DALYs) of gout, and discerning its burden trends, is of utmost urgency. Such insights are crucial for formulating targeted prevention and management strategies, ultimately enhancing health outcomes.

The Global Burden of Disease (GBD) database, established by the Global Burden of Disease Study and maintained by the Institute for Health Metrics and Evaluation (IHME) at the University of Washington, is a comprehensive repository that systematically quantifies and evaluates the impacts of major diseases, injuries, and health risk factors at global, regional, and national levels (9, 10). To date, no study has utilized the GBD database to specifically analyze the disease burden of gout in East Asian adolescents (aged 10–19 years) from 1990 to 2021.

This study leveraged GBD 2021 data to analyze incidence, prevalence and disability-adjusted life years (DALYs) of gout from 1990 to 2021 in adolescents (aged 10–19 years), in global level, East Asia, and countries and regions in East Asia. Then, the study identified disparities in gout burden across different gender groups. Furthermore, the study projected gout burden to 2050 using demographic and epidemiologic forecasting models, with a focus on levels of global, East Asia and China. The findings of this study contribute to the existing literature by providing valuable information for healthcare policies and resource allocation, as well as guiding the development of prevention and intervention strategies to effectively reduce the disease burden of gout.

## Materials and methods

### Study population and data collection

The GBD 2021 is the most comprehensive and detailed study of global diseases, injuries, and risk factors. It covers 204 countries and territories and estimates the burden of 371 diseases and injuries from 1990 to 2021, including gout. In the cross-sectional study, our data were collected from the GBD Study 2021 by using the Global Health Data Exchange (GHDx) query tool,^1^ involving adolescents with gout in global level, East Asia, countries and regions in East Asia, including China, Taiwan (Province of China), Japan, Republic of Korea, Democratic People’s Republic of Korea and Mongolia. Adolescents are defined as individuals aged 10–19 years according to the World Health Organization (WHO). The GBD database is classified based on the international classification of diseases (ICD) system. The code for gout is ICD-10-M10.

The study focused on the trends of following basic indicators: incidence, prevalence and disability-adjusted life years (DALYs). DALYs is a measure of disease burden, which combines years of life lost due to premature mortality and years lived with disability (YLDs). The study collected data from GBD 2021 on the incidence, prevalence, age-standardized incidence rates (per 100,000 persons), age-standardized prevalence rates (per 100,000 persons) and age-standardized DALYs rates (per 100,000 persons) of gout in 204 countries and territories from 1990 to 2021, classified by year and gender, with a special focus on countries and regions in East Asia. High body-mass index, kidney dysfunction, and metabolic risks were extracted from the GBD Results Tool as the only three level 2 risks associated with DALYs, which were included as three potential risk factors for gout in the study.

Sociodemographic index (SDI), ranging from 0 to 1, is a comprehensive measurement of education, economic, and fertility levels. Higher SDI values represent higher levels of socioeconomic development. SDI is divided into five quintiles: low, low-middle, middle, high-middle, and high. The relation between the DALYs of gout and SDI for different GBD regions were further analyzed with Smoothing Splines model.

Risk factors and their definitions were derived from the Global Burden of Disease (GBD) 2021 study, which systematically evaluates 88 risk factors across 631 risk–outcome pairs based on evidence. For each risk–outcome pair, relative risks (RRs) were meta-analyzed from cohort studies and clinical trials, and population attributable fractions (PAFs) were calculated using following formula:

$$\mathrm{PAF}=\frac{\sum_{i=1}^{n}P_{i}\cdot {\mathrm{RR}}_{i}-\sum_{i=1}^{n}P_{i}\cdot {\mathrm{RR}}_{i}^{\text{TMREL}}}{\sum_{i=1}^{n}P_{i}\cdot {\mathrm{RR}}_{i}}$$

Mediation adjustments were applied for risks acting indirectly through intermediate factors. The percentage contributions in our results reflect GBD-calculated PAFs, representing the proportion of gout DALYs attributable to each risk factor (11). The proportion of DALYs for gout attributable to potential risk factors in different genders was analyzed, respectively, in the study. As all GBD data used in the study were from publicly available databases, further ethical approval was not required.

### Statistical analysis

Firstly, the estimated annual percentage change (EAPC) was used to quantify the overall trend of gout burden. The EAPC derived from a linear regression model (12). If the EAPC estimation and its lower boundary of 95% CI were both > 0, the ASR was recognized to be in an increasing trend. On the contrary, if the EAPC estimation and its upper boundary of 95% CI were both < 0, the ASR was recognized to be in a decreasing trend. Otherwise, the ASR was stable. The Autoregressive Integrated Moving Average model (ARIMA) was used to predict the age-standardized rate of DALYs in gout from 2022 to 2050, for global level, East Asia, and China, respectively. We applied frontier analysis to further assess the relationship between the gout burden and sociodemographic development. All statistical analyses and graphics were executed using R version 4.3.3.

## Results

### Overall gout burden in adolescents in level of global, East Asia and China

In global level, 22.7 thousand prevalent cases of gout were reported among adolescents aged 10–19 years in 2021, with an age-standardized rate (ASR) of 653.8 per 100,000, reflecting an increase of 21.9% since 1990. In 2021, 15.8 thousand incident cases of gout were reported globally among adolescents, with an ASR of 109.1 per 100,000, reflecting an increase of 23.4% since 1990. The number of DALYs for gout globally among adolescents was 0.80 thousand, with an ASR of 20.2 DALYs per 100,000, a 21.3% increase since 1990 (Table 1). The global gout disease burden in adolescents demonstrated an increasing trend, with the ASR of incidence increased by 0.26% annually, the ASR of prevalence increased by 0.26% annually and the ASR of DALYs increased at 0.26% annually (Table 2). In East Asia, 3.87 thousand prevalent cases, 1.58 incident cases and 0.14 thousand DALYs of gout were reported among adolescents in 2021 (Table 1). In East Asia, the gout burden showed a similar increasing trend in adolescents, with the ASR of incidence increased by 0.36% annually, the ASR of prevalence increased by 0.37% annually and the ASR of DALYs increased at 0.37% annually (Table 2). In China, 3.66 thousand prevalent cases, 2.55 incident cases and 0.13 thousand DALYs of gout were reported among adolescents in 2021 (Table 1), with the ASR of incidence, prevalence and DALYs all increased by 0.32% annually (Table 2).

**Table 1:** Prevalent cases, incident cases, disability-adjusted life years (DALYs) and percentage change in age-standardized rates (ASRs) per 100,000 for gout, in adolescents aged 10–19 years in 2021, by global, East Asia and countries and regions in East Asia, by Global Burden of Disease region from 1990 to 2021 (generated from data available at https://ghdx.healthdata.org/gbd-results-tool).

Countries and regions	Prevalence (95%UI)			Incidence (95% UI)			DALYs (95% UI)		
	No (95%UI)	ASRs per 100,000 (95% UI)	Percentage change in ASRs from 1990 to 2021	No (95%UI)	ASRs per 100,000 (95% UI)	Percentage change in ASRs from 1990 to 2021	No (95%UI)	ASRs per 100,000 (95% UI)	Percentage change in ASRs from 1990 to 2021
Global	22689.03 (7092.62, 50080.48)	653.8 (526.1, 810.5)	21.9 (20.3, 23.2)	15831.73 (4974.61, 34058.74)	109.1 (86.4, 135.8)	17.2 (15.7, 18.4)	796.38 (228.72, 1789.17)	20.2 (13.8, 28.8)	21.3 (19.5, 23.1)
East Asia	3873.96 (1348.41, 8298.32)	814.7 (649.4, 1,014)	26.2 (23.6, 28.8)	2696.73 (953.51, 5581.02)	151.9 (121.6, 189.5)	23.4 (21.3, 25.6)	135.97 (43.19, 309.36)	25.6 (17.3, 36.4)	25.7 (22.4, 29.6)
People’s Republic of China	3657.26 (1251.67, 7860.67)	810.4 (644.8, 1009.1)	25.1 (17.4, 36.6)	2546.72 (894.59, 5293.7)	151.6 (121.2, 189.2)	17.1 (9.1, 25.8)	128.35 (40.76, 291.28)	25.4 (17.2, 36.3)	24.9 (11.9, 41.1)
Taiwan (Province of China)	110.24 (41.91, 228.66)	1054.1 (865.3, 1295.6)	19.9 (12.7, 27.1)	76.04 (28.76, 156.88)	177.3 (142.3, 218.4)	14 (7.7, 19.4)	3.88 (1.33, 8.12)	33.2 (22.8, 48)	20.2 (6.1, 36.2)
Democratic People’s Republic of Korea	106.47 (38.31, 226.22)	793 (631, 982.7)	26.5 (23.7, 29.1)	73.97 (27.3, 155.94)	143 (112.6, 176.8)	23.7 (21.7, 26)	3.74 (1.27, 8)	25.1 (16.7, 36.2)	25.9 (22.5, 30.2)
Japan	203.9 (59.4, 437.17)	722.9 (568.2, 917)	14.8 (8.3, 21.4)	141.71 (42.15, 299.02)	116.7 (91.9, 147.5)	12.4 (6.2, 18.7)	7.16 (2.02, 15.85)	22.7 (15.4, 32.7)	14.9 (2.6, 28.9)
Mongolia	7.06 (1.71, 15.43)	415.7 (331, 514.5)	11.5 (9.7, 13.2)	4.93 (1.22, 10.63)	79.4 (63.5, 100.2)	8.7 (7.4, 10.1)	0.25 (0.06, 0.57)	12.9 (8.5, 18.8)	11.9 (8.7, 14.6)
Republic of Korea	95% UI = 95% uncertainty intervals. 89.98 (27.36, 195.99)	728.6 (571.9, 919.2)	9.2 (2.9, 15.4)	62.46 (18.83, 135.7)	107.2 (84, 134)	8.1 (2.3, 14.4)	3.16 (0.93, 7.26)	22.7 (15.2, 33)	8.4 (−6.1, 25.3)

95% UI, 95% uncertainty intervals.

**Table 2:** The EAPC in incidence, prevalence and DALYs for gout, in adolescents aged 10–19 years by global, East Asia and countries and regions in East Asia, from 1990 to 2021 (generated from data available at https://ghdx.healthdata.org/gbd-results-tool).

Countries and regions	EAPC in ASRs of incidence (95% CI)	EAPC in ASRs of prevalence (95% CI)	EAPC in ASRs of DALYs (95% CI)
People’s Republic of China	0.32 (−0.04, 0.67)	0.32 (−0.03, 0.68)	0.32 (−0.03, 0.68)
Democratic People’s Republic of Korea	0.62 (0.52, 0.72)	0.63 (0.53, 0.72)	0.63 (0.53, 0.72)
Japan	−0.06 (−0.11, −0.01)	−0.06 (−0.11, −0.01)	−0.06 (−0.11, −0.01)
Mongolia	0.34 (0.12, 0.56)	0.34 (0.11, 0.56)	0.33 (0.11, 0.56)
Republic of Korea	0.49 (0.28, 0.7)	0.49 (0.28, 0.7)	0.49 (0.28, 0.7)
Taiwan (Province of China)	1.61 (1.14, 2.09)	1.62 (1.14, 2.11)	1.63 (1.15, 2.12)
East Asia	0.36 (0.03, 0.69)	0.37 (0.03, 0.7)	0.37 (0.04, 0.7)
Global	0.26 (0.12, 0.4)	0.26 (0.12, 0.4)	0.26 (0.12, 0.4)

DALYs, disability-adjusted life years.

All countries and regions in East Asia showed an increasing trend in disease burden of gout among adolescents in 2021. Taiwan (Province of China) had the highest age-standardized rates of prevalence, incidence and DALYs for gout (1054.1, 177.3, 33.2 per 100,000 respectively), whereas Mongolia had the lowest in all three indicators (415.7, 79.4, 12.9 per 100,000 respectively) (Table 1). From 1990 to 2021, the largest increase in the age-standardized point prevalence, incidence and DALY rates of gout was found in Democratic People’s Republic of Korea (+26.5%, +23.7%, +25.9% respectively) (Table 1). China showed the second largest increase in the prevalence, incidence and DALY rates of gout (+25.1%, +17.1%, +24.9% respectively) (Table 1). The smallest increase in the age-standardized point prevalence, incidence and DALY rates of gout was found in Republic of Korea (+9.2%, +8.1%, +8.4% respectively) (Table 1).

**Figure 1:**
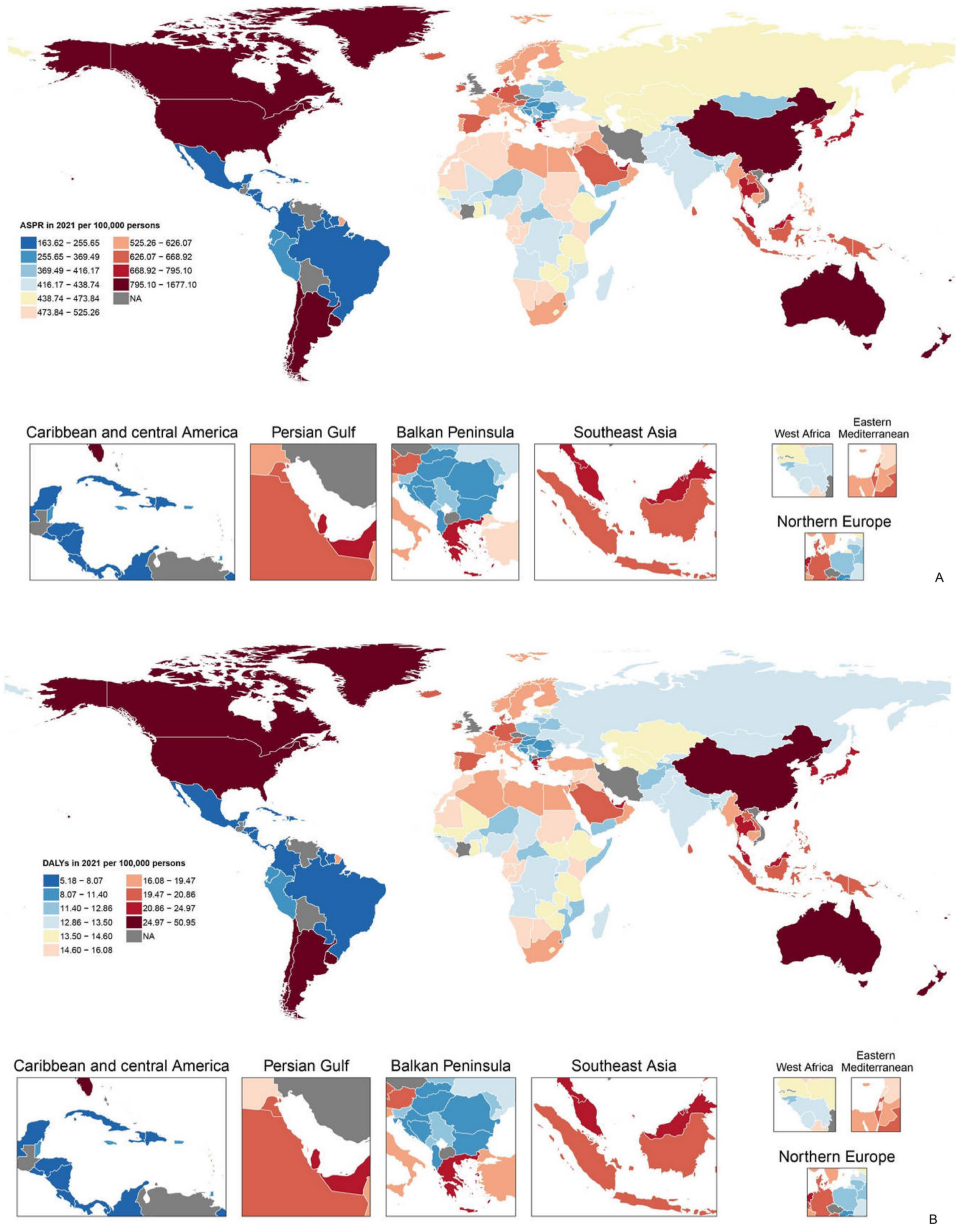
**(A)** Age-standardized point prevalence of gout per 100,000 population in 2021 by countries and **(B)** age-standardized DALYs of gout per 100,000 population in 2021 by countries (generated from data available at https://ghdx.healthdata.org/gbd-results-tool). Map created in R using r-maps package (https://cran.r-project.org/web/packages/maps/index.html). DALYs, disability-adjusted life years.

The trends of disease burden of gout varied considerably across 204 countries and territories (Figure 1). The highest ASR of prevalence was found in United States of America and the lowest in Guatemala (Figure 1A); Similarly, the highest ASR of DALYs was found in United States of America and the lowest in Guatemala (Figure 1B). Sex differences in gout burden.

**Figure 2:**
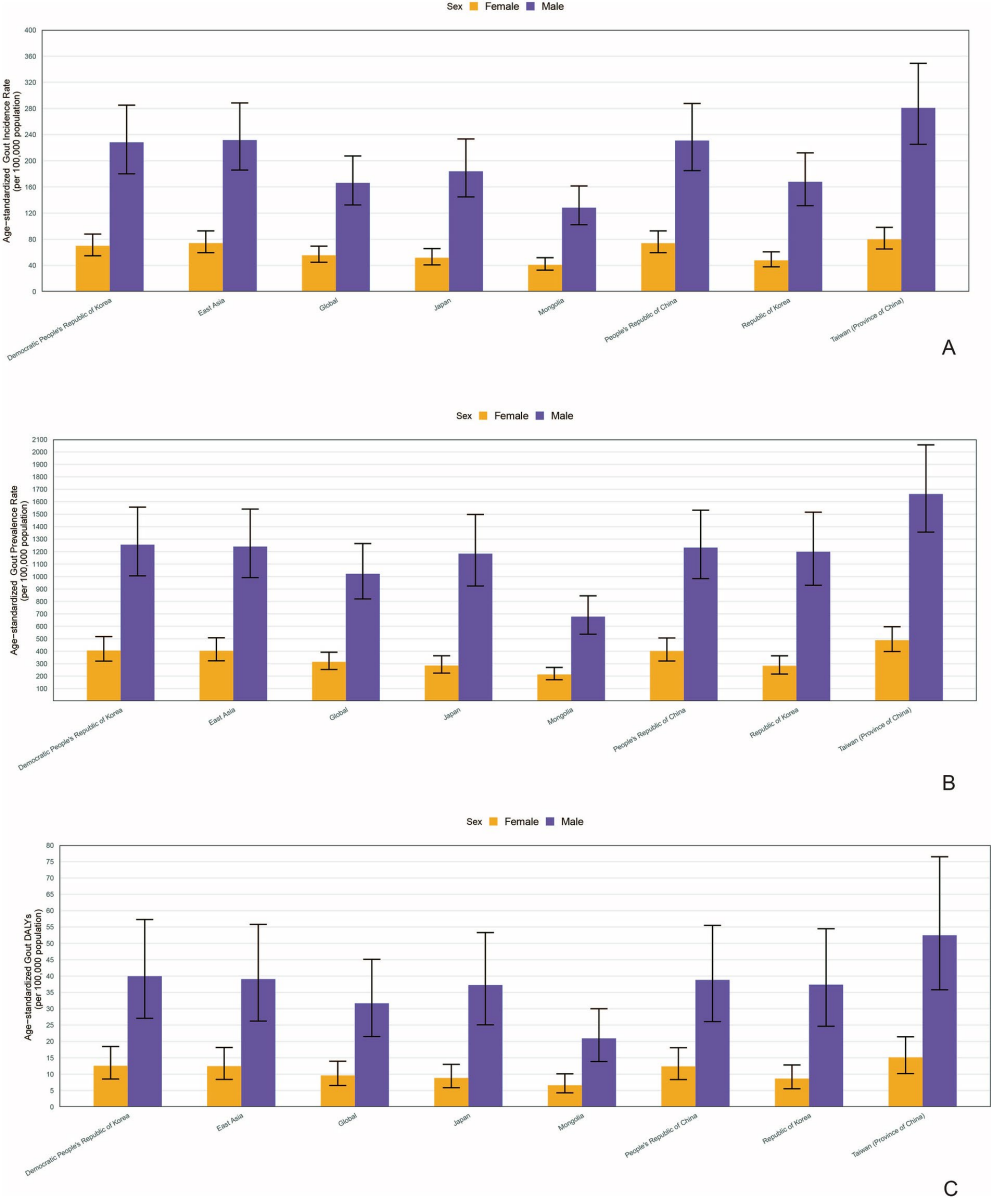
The age-standardized rate (ASR) of incidence **(A)**, prevalence **(B)** and DALY **(C)** in different genders in countries and regions in East Asia (generated from data available at https://ghdx.healthdata.org/gbd-results-tool). DALYs, disability-adjusted life years.

In 2021, the number of incident cases, prevalent cases and DALYs in adolescents was significantly higher in females in all countries and regions in East Asia (Supplementary Figures S1A–C). Similarly, the age-standardized rate (ASR) of incidence, prevalence and DALY all showed a higher value in females in all countries and regions in East Asia (Figures 2A–C).

### Association with the sociodemographic index

**Figure 3:**
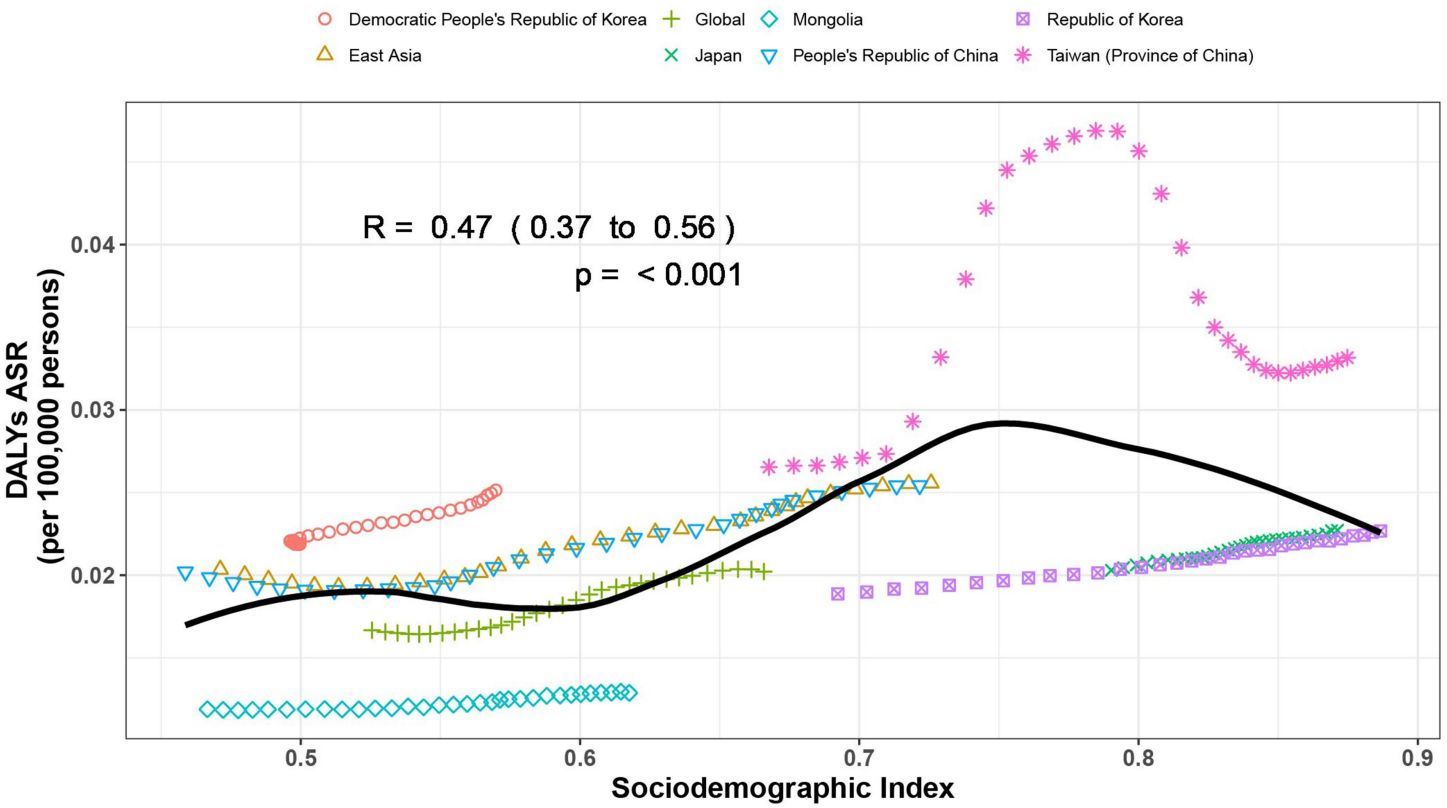
Age-standardized disability-adjusted life year (DALY) rates of gout by sociodemographic index (SDI), by global, East Asia and countries and regions in East Asia, from 1990 to 2021. Thirty points are plotted for each region and show the observed age-standardized DALY rates from 1990 to 2021 for that region. Expected values, based on sociodemographic index and disease rates in all locations, are shown as a solid black line. Regions above the solid line represent a higher-than-expected burden and regions below the line show a lower-than-expected burden (generated from data available at https://ghdx.healthdata.org/gbd-results-tool).

We found a M shaped association between the sociodemographic index and the age-standardized DALY rate of gout from 1990 to 2021, in level of global, East Asia, and countries and regions in East Asia. The age-standardized DALY rate basically maintained an upward trend until the SDI increased to around 0.75. When the SDI exceeded 0.75, with the growth of SDI, the age-standardized DALY rate showed a declining trend. Taiwan (Province of China) and Democratic People’s Republic of Korea had higher-than-expected DALY rates based on their sociodemographic index from 1990 to 2021. In contrast, Mongolia and Japan had lower-than-expected-burdens from 1990 to 2021 (Figure 3).

### Risk factors for gout in adolescents

**Figure 4:**
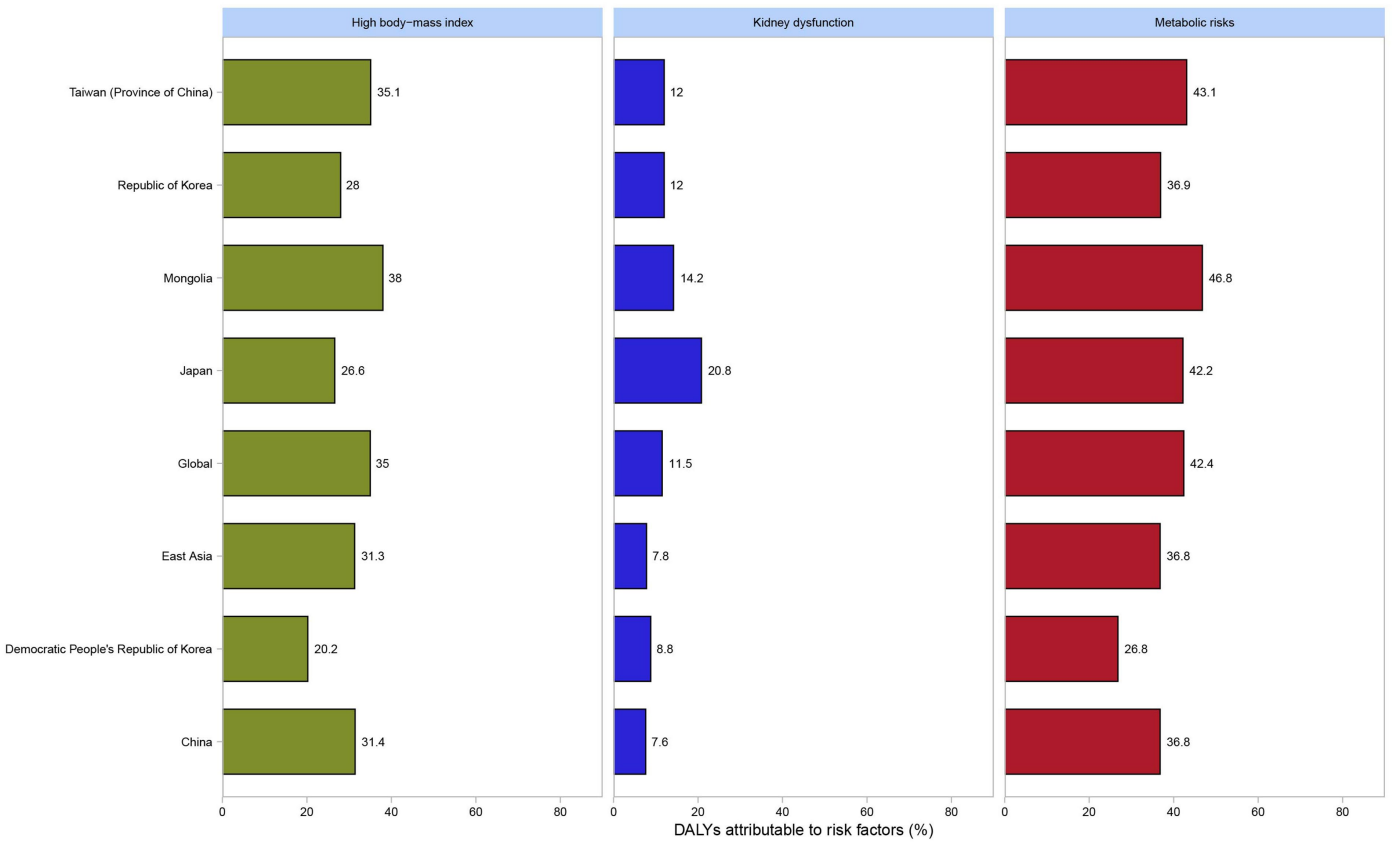
Percentage of DALYs due to gout attributable to high body-mass index, kidney dysfunction and metabolic risks among both genders by global, East Asia and countries and regions in East Asia in 2021 among adolescents (generated from data available from http://ghdx.healthdata.org/gbd-results-tool). DALYs, disability-adjusted life years.

In 2021, high body-mass index, kidney dysfunction, and metabolic risks were three potential risk factors contributing to gout in adolescents. Globally, among both genders, the contribution proportion of metabolic risk to DALYs was 42.4%, that of a high body-mass index was 35%, and that of renal dysfunction was 11.5%. In East Asia, the proportion of metabolic risk was 36.8%, that of a high body-mass index was 31.3%, and that of renal dysfunction was 7.8%. Similar to the situation in East Asia, in China, the proportions of DALYs attributable to metabolic risk, a high body-mass index, and renal dysfunction were 36.8%, 31.4%, and 7.6%, respectively (Figure 4). Metabolic risks were the most significant risk factor contributing to DALYs of gout in all countries and regions in East Asia. Among them, Mongolia had the highest proportion at 46.8%, while the Democratic People’s Republic of Korea had the lowest at 26.8%. In contrast, kidney dysfunction was the least significant risk factor contributing to DALYs of gout in all countries and regions in East Asia. Japan had the highest proportion in this regard at 20.8%, and China had the lowest at 7.6% (Figure 4). When analyzed separately for males and females, metabolic risks were the predominant factor contributing to DALYs of gout, followed by high body-mass index. Globally and across all countries and regions in East Asia, the percentages of DALYs attributable to the three risk factors were consistently higher among females than among males (Supplementary Figure S2).

### Predictive analysis on gout burden to 2050

**Figure 5:**
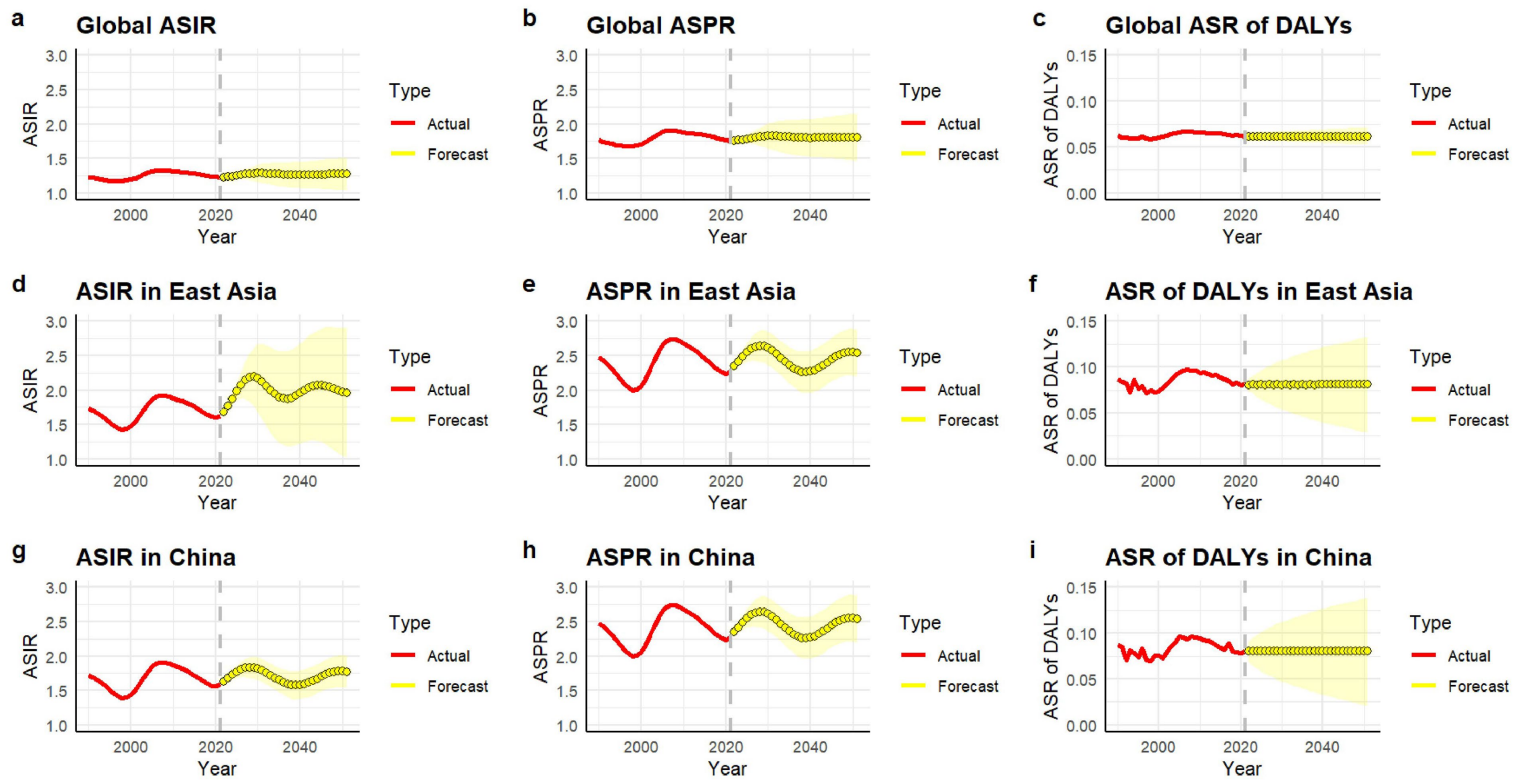
ASIR **(a,d,g)**, ASPR **(b,e,h)**, and ASR of DALYs **(c,f,i)** (per 100,000 persons) of gout predicted from 2022 to 2050 by global, East Asia and China, respectively, using ARIMA forecasting. ASIR, age-standardized incidence rate; ASPR, age-standardized prevalence rate; DALY, disability-adjusted life years.

At the global level, the predicted values of the age-standardized incidence rate (ASIR) in 2022–2050 were basically stable with extremely small fluctuations. The predicted values of the age-standardized prevalence rate (ASPR) and the age-standardized rate of disability-adjusted life years (ASR of DALYs) were also relatively stable (Figures 5a–c). At the level of East Asia, the predicted values of ASIR and ASPR fluctuated greatly and showed an upward trend, while the ASR of DALYs was relatively stable (Figures 5d–f). Similar with East Asia, in China, the predicted values of ASIR and ASPR fluctuated greatly and showed an upward trend, while the ASR of DALYs was relatively stable (Figures 5g–i).

### Frontier analysis of age-standardized DALYs rates

**Figure 6:**
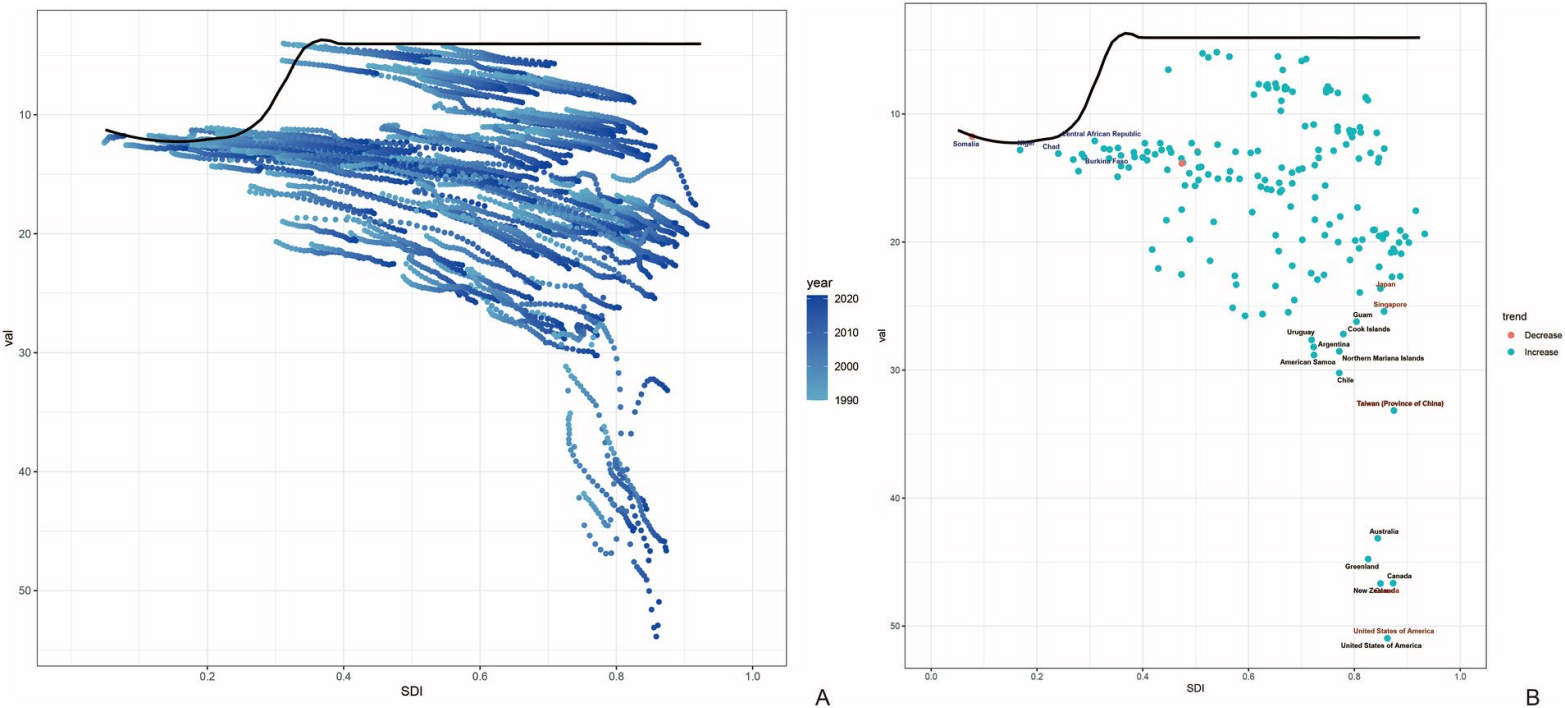
Frontier analysis based on SDI and age-standardized DALY rates of gout from 1990 to 2021. The frontier is delineated in solid black color; countries and territories are represented as dots **(A)**. The top 15 countries with the largest effective difference (largest gout DALYs gap from the frontier) are labeled in black; examples of frontier countries with low SDI (<0.5) and low effective difference are labeled in blue (e.g., Somalia, Nigeria, Chad, Central African Republic and Burkina Faso), and examples of countries and territories with high SDI (>0.85) and relatively high effective difference for their level of development are labeled in red (e.g., Japan, Singapore, Taiwan (Province of China), United States of America, and Canada). Red dots indicate an increase in age-standardized DALYs rate from 1990 to 2021; blue dots indicate a decrease in age-standardized DALYs rate between 1990 and 2021 **(B)**. SDI, socio-demographic index; DALYs, disability-adjusted life years.

**Figure 7:**
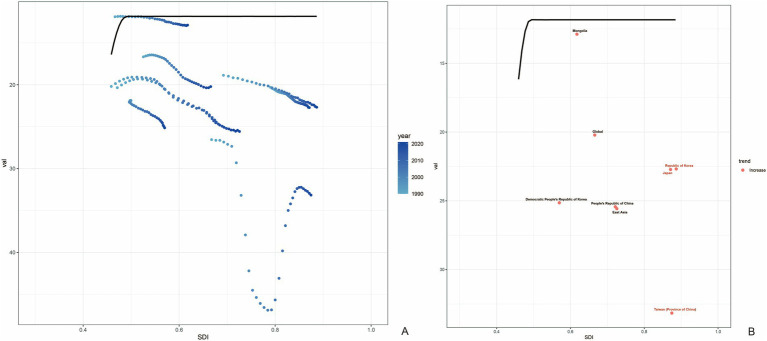
Frontier analysis based on SDI and age-standardized DALY rates of gout in countries and regions in East Asia from 1990 to 2021. The frontier is delineated in solid black color; countries and territories are represented as dots **(A)**. Countries and regions with high SDI (>0.85) and relatively high effective difference for their level of development are labeled in red (Japan, Republic of Korea and Taiwan (Province of China)) **(B)**.

The frontier analysis based on SDI and age-standardized DALY rates of gout spanning from 1990 to 2021 across 204 countries and territories revealed a distinct trend. The age -standardized rates of DALY increased rapidly when the SDI value rose from 0.3 to 0.4, while remaining relatively stable at other SDI values (Figure 6A). Figure 6B showed the DALYs gout burden and the effective difference in countries or regions with different sociodemographic development levels in 2021. In most countries and regions (represented by green dots), the age-standardized DALY rates increased as the SDI value rose. The frontier analysis based on SDI and age-standardized DALY rates of gout spanning from 1990 to 2021 across countries and regions in East Asia was shown in Figure 7. The age -standardized rates of DALY increased when the SDI value rose from 0.45 to 0.5, then remained stable as the SDI increased. In all countries and regions in East Asia, the age-standardized DALY rates increased as the SDI value rose (Supplementary Table S1). Japan, Republic of Korea and Taiwan (Province of China) had high SDI (>0.85) and relatively high effective difference for their level of development (Figure 7B).

## Discussion

To our knowledge, this was the first study to describe the gout burden and its changing trends among adolescents aged 10–19 years globally, in East Asia, and in China from 1990 to 2021, based on the data of the Global Burden of Disease (GBD) in 2021. Moreover, we utilized a predictive model to deduce the evolving trends of the disease burden of gout from 2022 to 2050. The study revealed the multi-dimensional characteristics of the disease burden of gout among adolescents, offering critical insights for public health planning and clinical interventions.

Our analysis demonstrated a significant and consistent upward trend in the disease burden of gout among adolescents globally, in East Asia, and specifically in China from 1990 to 2021, aligning with previous studies on global gout burden in recent years (5, 7, 8, 13). This trend is underpinned by a complex interplay of biological, genetic, and environmental factors uniquely prominent in young populations. Lifestyle changes triggered by rapid socioeconomic development, such as increased consumption of high-purine diets and decreased physical activity levels, likely drove to this rise (3, 14, 15). Critically, adolescents with gout exhibit distinct pathogenic features compared to adults, including significantly higher serum urate levels, stronger genetic predisposition and higher prevalence of underexcretion-type hyperuricemia (16, 17). Whole-genome sequencing studies further identify novel genetic loci (RCOR1, FSTL5-MIR4454) and transporter variants (ABCG2, SLC22A12) associated with early-onset gout, with RCOR1 promoting NLRP3 inflammasome activation and IL-1β release, which are key drivers of gouty inflammation (18).

Within East Asia, there was notable heterogeneity in the growth of the disease burden of adolescent gout across countries and regions. For instance, Democratic People’s Republic of Korea showed the most significant increases in age-standardized prevalence, incidence, and disability-Adjusted Life Years (DALYs) rates, while the increasement in ASRs in Republic of Korea remained relatively low. This discrepancy could be likely attributed to different socioeconomic development levels and accessibility to healthcare resources. Noticeably, Taiwan (Province of China) had the highest age-standardized rates of prevalence, incidence and DALYs for gout among countries and regions in East Asia. Previous studies have shown that genetic factors among the population in Taiwan (Province of China) have led to a high proportion of patients with early-onset gout (EOG), particularly ABCG2 dysfunction (18), which may explain why the ASRs of gout in Taiwan (Province of China) was the highest in East Asia (19).

In China, the upward trend of the disease burden of adolescent gout was a cause for concern. With the acceleration of the urbanization process and the increase in income, the structure of diet for adolescents has developed towards a high-purine diet including sugary beverages, red meat, and seafood (20). Meanwhile, in recent years, the obesity rate and the prevalence rate of metabolic syndrome among Chinese adolescents have increased significantly due to lack of physical activity and increasing sedentary time (21–23). These risk factors collectively formed an important basis for the onset of gout.

Moreover, the reason for the increase in prevalence in recent years may also due to the change in diagnostic criteria. In 2015, the American College of Rheumatology (ACR) and the European League against Rheumatism (EULAR) jointly issued a new diagnostic classification for gout. The 2015 ACR-EULAR gout classification criteria showed a significantly better diagnostic performance and allowed earlier diagnosis for gout compared to the 1977 ACR criteria, which may induce to the increasing of gout prevalence (24–27), potentially capturing more adolescent patients with atypical presentations (16).

In 2021, the age-standardized rates of prevalence, incidence and DALYs of gout among male adolescents in East Asia were significantly higher than those among female adolescents. This result was consistent with previous studies, indicating that males also had a higher susceptibility to gout during adolescence (4, 7, 28). Additionally, study showed that young males aged 17–19 exhibited higher baseline urate levels (17). This might be because estrogen and progesterone could promote uric acid excretion, reducing the risk of hyperuricemia and gout in females (29). Beyond hormonal influences, non-biological factors related to gender differences may also contribute to disparities in gout burden. In several East Asian countries, alcohol consumption rates and total intake are substantially higher among men than women (30–32). Studies have shown that alcohol consumption is associated with an elevated risk of gout (33). Additionally, gender-based imbalances in healthcare access and socioeconomic status may lead to diagnostic bias in gout, though future research is needed to verify this. It’s worth noting that in recent years, the prevalence of gout among females globally and in East Asia had been continuously increasing. Some studies even showed that the annual increase in the prevalence of gout in the United States was only observed among females. We speculated that this might be related to the increase in obesity, high-purine diets, and unhealthy lifestyles among females, as well as the lower proportion of females receiving uric acid-lowering treatment (34, 35). Moreover, women were more often to have renal disease and concomitant use of thiazides or other diuretics, which might increase the risk of kidney dysfunction, thus providing risk factors for gout (36). Therefore, we should pay more attention to the prevention and control of gout in women.

The Social Demographic Index (SDI) showed a non-linear “M”-shaped association pattern with the age-standardized Disability-Adjusted Life Years (DALYs) rate of gout. When the SDI value was lower than 0.75, the age-standardized DALYs rate showed an upward trend. After exceeding this threshold, with the further increase of the SDI value, the disease burden showed a declining trend. The non-linear relationship with SDI suggests complex interactions between development, lifestyle changes, healthcare access, and gout burden that evolve across different socioeconomic stages. In the initial stage of development, the rapid transformation of lifestyle was likely to exacerbate the disease risk. While at the stage of high development level, factors such as the high-quality medical service system and the improvement of public health literacy played an important protective role.

High body-mass index, kidney dysfunction, and metabolic risks were three potential risk factors contributing to gout included in GBD 2021 data. Metabolic risks emerged as the predominant factor across all East Asian countries and regions for both genders, contributing the largest proportion to gout DALYs (e.g., 36.8% in East Asia, 36.8% in China). High BMI was the second major contributor (e.g., 31.3% in East Asia, 31.4% in China), followed by kidney dysfunction (e.g., 7.8% in East Asia, 7.6% in China). Significant variation existed across East Asia: Mongolia had the highest proportion attributed to metabolic risks (46.8%), while the Democratic People’s Republic of Korea had the lowest (26.8%). Conversely, Japan had the highest proportion attributed to kidney dysfunction (20.8%), and China the lowest (7.6%). This might be related to the high prevalence of kidney dysfunction previously reported in high-income Asia-Pacific regions (37). Previous studies have demonstrated that individuals with obesity (BMI > 30 kg/m^2^) have over twice the risk of developing gout compared to those with a BMI < 30 kg/m^2^, which aligned with the identification of high body-mass index as a significant risk factor for gout (38). A critical finding was that the percentage contributions of all three risk factors (metabolic risks, high BMI, and kidney dysfunction) to gout DALYs were significantly higher among adolescent females compared to males, both globally and within every East Asian country and region analyzed (Supplementary Figure S2), suggesting that females may exhibit greater susceptibility to the gout-promoting effects of these metabolic and renal factors per unit of exposure. This finding underscored the importance of considering gender-specific pathways in understanding gout etiology and tailoring prevention strategies. The exceptionally high gout burden observed in Taiwan (Province of China) warranted specific consideration of risk factors. While our analysis confirmed the dominance of metabolic risks in Taiwan (Province of China), early genetic studies suggest a potential predisposition to early-onset gout (EOG) in this population (19). The interplay between genetic susceptibility and metabolic risks identified in Taiwan (Province of China) likely created a perfect storm contributing to its leading position in adolescent gout burden in East Asia. The predictive analysis showed that the age-standardized rates of incidence, prevalence and disability-Adjusted Life Years (DALYs) of gout among adolescents globally would remain relatively stable by 2050. In contrast, the age-standardized rates of incidence and prevalence of gout among adolescents in East Asia and in China were expected to exhibit significant fluctuations and continue to rise, while the ASRs of DALYs would remain relatively stable. The prediction results indicated that in the coming decades, East Asia and China needed to continuously strengthen the construction of the prevention and control system for adolescent gout to effectively address the potential increase in the disease burden, including genetic screening for high-risk adolescents, obesity interventions, and improved ULT adherence programs.

This study had limitations. First, GBD 2021 data reliance introduced potential bias from variable quality and limited cross-regional comparability, especially in low-resource settings. Second, restricting analysis to three GBD risk factors (high BMI, kidney dysfunction and metabolic risks) precluded assessing established contributors like purine-rich diets and genetic susceptibility due to data heterogeneity and lack of sufficient standardized global data for GBD 2021. Third, secondary data prevented validation of diagnostic accuracy, disease severity and treatment adherence. Fourth, observed male predominance in gout burden required controlling for unmeasured confounders including healthcare access disparities, diagnostic biases, hormonal influences and comorbidity profiles. Fifth, differential healthcare systems, cultural perceptions, and data collection methods across East Asia may skew trend interpretations. Finally, although ARIMA models project rising gout burden through 2050, these estimates assumed current trends persisted and lacked external validation.

## Conclusion

In conclusion, this study revealed an increasing gout burden in global, East Asia and China among adolescents with significant gender disparities and distinct regional variations within East Asia based on GBD 2021 data. Critically, projections indicate the gout burden in East Asia and China is expected to continue rising towards 2050. Efforts should be focused on strengthening education regarding adherence to gout treatment and controlling relevant risk factors to reduce the overall gout burden in East Asia and China.

## Data Availability

The original contributions presented in the study are included in the article/Supplementary material, further inquiries can be directed to the corresponding author.

## Footnotes

^1^ http://ghdx.healthdata.org/gbd-results-tool
