# Supplementary material for: Disease burden and trends in gout for adolescents from 1990 to 2021, with projections to 2050 globally, in East Asia and China: results from the Global Burden of Disease study 2021
Source: Front Public Health. 2025 Aug 13;13:1629891. doi: 10.3389/fpubh.2025.1629891 (PMC12380837; doi:10.3389/fpubh.2025.1629891)
Supplement: Supplementary file 1 [file Data_Sheet_1.docx]

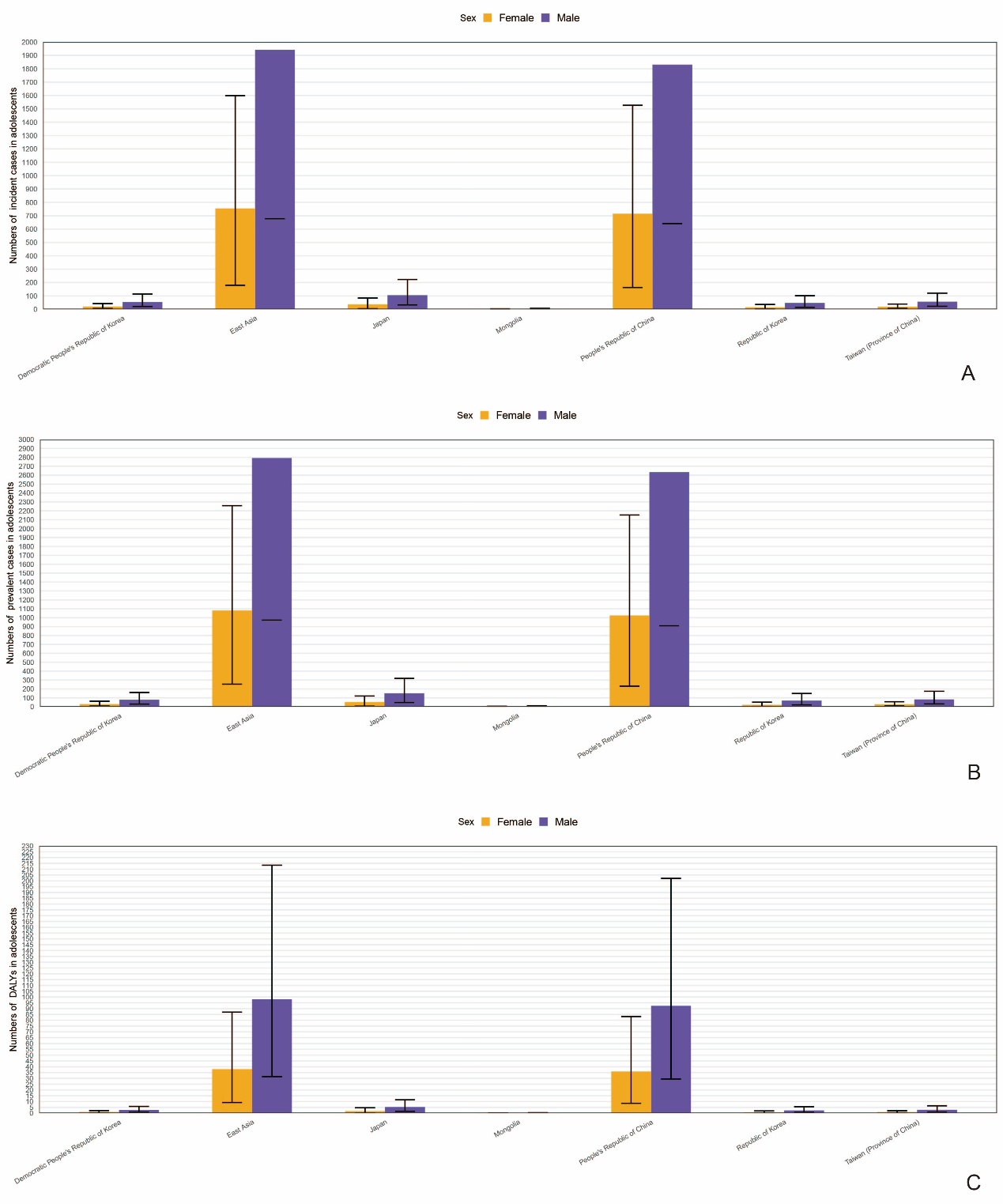


Fig S1 | The number of incident cases (A), prevalent cases (B) and DALYs (C) in adolescents in countries and regions in East Asia in 2021 (generated from data available at <https://ghdx.healthdata.org/gbd-results-tool>). DALYs: disability-adjusted life years;


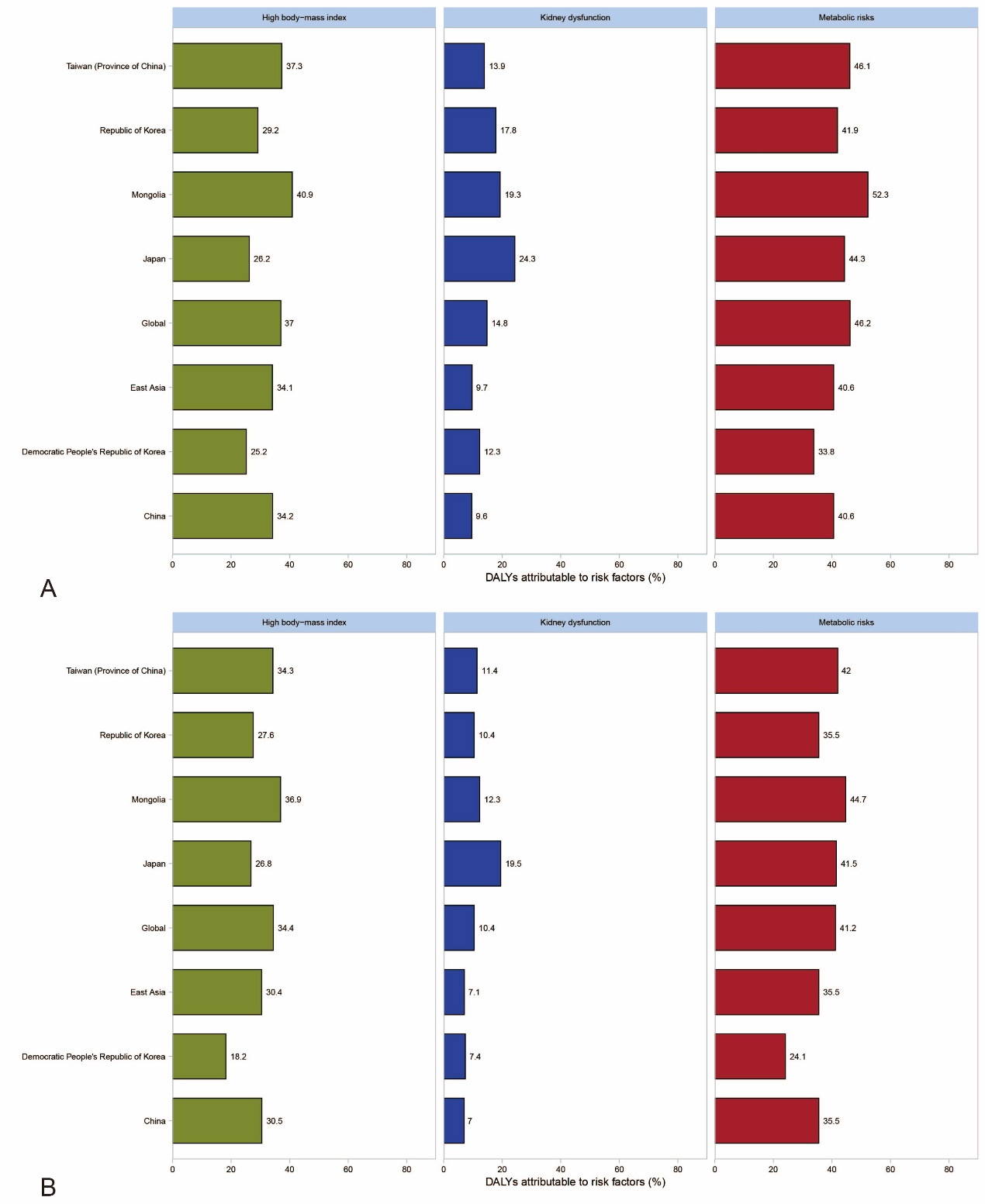


Fig S2| Percentage of DALYs due to gout attributable to high body-mass index, kidney dysfunction and metabolic risks among females (A) and males (B) by global, East Asia and countries and regions in East Asia in 2021 (Generated from data available from http://ghdx.healthdata.org/gbd-results-tool). DALYs: disability-adjusted life years;

|  | year | val | SDI | frontier | eff_diff | trend |
| --- | --- | --- | --- | --- | --- | --- |
| Democratic People's Republic of Korea | 2021 | 25.13979 | 0.5698546 | 11.83582 | 13.303962 | Increase |
| East Asia | 2021 | 25.57028 | 0.7257049 | 11.83764 | 13.732634 | Increase |
| Global | 2021 | 20.21783 | 0.665821 | 11.83402 | 8.38381 | Increase |
| Japan | 2021 | 22.72161 | 0.8712418 | 11.83639 | 10.885212 | Increase |
| Mongolia | 2021 | 12.88416 | 0.6176216 | 11.83392 | 1.050233 | Increase |
| People's Republic of China | 2021 | 25.43052 | 0.7216298 | 11.8347 | 13.595819 | Increase |
| Republic of Korea | 2021 | 22.68012 | 0.8866753 | 11.83342 | 10.846702 | Increase |
| Taiwan (Province of China) | 2021 | 33.16027 | 0.8747471 | 11.83481 | 21.325456 | Increase |

Table S1| Frontier analysis based on SDI and age-standardized DALY rate of gout in 2021, by global, East Asia and countries and regions in East Asia (Generated from data available from http://ghdx.healthdata.org/gbd-results-tool). DALYs: disability-adjusted life years;
